# Proliferative potential and resistance to immune checkpoint blockade in lung cancer patients

**DOI:** 10.1186/s40425-019-0506-3

**Published:** 2019-02-01

**Authors:** Sarabjot Pabla, Jeffrey M. Conroy, Mary K. Nesline, Sean T. Glenn, Antonios Papanicolau-Sengos, Blake Burgher, Jacob Hagen, Vincent Giamo, Jonathan Andreas, Felicia L. Lenzo, Wang Yirong, Grace K. Dy, Edwin Yau, Amy Early, Hongbin Chen, Wiam Bshara, Katherine G. Madden, Keisuke Shirai, Konstantin Dragnev, Laura J. Tafe, Daniele Marin, Jason Zhu, Jeff Clarke, Matthew Labriola, Shannon McCall, Tian Zhang, Matthew Zibelman, Pooja Ghatalia, Isabel Araujo-Fernandez, Arun Singavi, Ben George, Andrew Craig MacKinnon, Jonathan Thompson, Rajbir Singh, Robin Jacob, Lynn Dressler, Mark Steciuk, Oliver Binns, Deepa Kasuganti, Neel Shah, Marc Ernstoff, Kunle Odunsi, Razelle Kurzrock, Mark Gardner, Lorenzo Galluzzi, Carl Morrison

**Affiliations:** 1 OmniSeq, Inc., 700 Ellicott Street, Buffalo, NY 14203 USA; 2 Roswell Park Comprehensive Cancer Center, Elm and Carlton Streets, Buffalo, NY 14206 USA; 3 Dartmouth-Hitchcock Medical Center, Lebanon, NH 03756 USA; 4 Duke University, Durham, NC 27708 USA; 5 Fox Chase Cancer Center, Philadelphia, PA 19111 USA; 6 Hospital Universitario Virgen Macarena, 41009 Sevilla, Spain; 7 Medical College of Wisconsin, Milwaukee, WI 53226 USA; 8 Meharry Medical College, Nashville, TN 37208 USA; 9 Mission Health System, Asheville, NC 28801 USA; 10 Community Hospital, Munster, IN 46321 USA; 11 Center for Personalized Cancer Therapy, Moores Cancer Center, La Jolla, CA 92093 USA; 12 Department of Radiation Oncology, Weill Cornell Medical College, New York, NY 10065 USA; 13 Sandra and Edward Meyer Cancer Center, New York, NY 10065 USA; 14 Université Paris Descartes/Paris V, 75006 Paris, France

**Keywords:** Atezolizumab, Nivolumab, Pembrolizumab, Ipilimumab, PD-1

## Abstract

**Background:**

Resistance to immune checkpoint inhibitors (ICIs) has been linked to local immunosuppression independent of major ICI targets (e.g., PD-1). Clinical experience with response prediction based on PD-L1 expression suggests that other factors influence sensitivity to ICIs in non-small cell lung cancer (NSCLC) patients.

**Methods:**

Tumor specimens from 120 NSCLC patients from 10 institutions were evaluated for PD-L1 expression by immunohistochemistry, and global proliferative profile by targeted RNA-seq.

**Results:**

Cell proliferation, derived from the mean expression of 10 proliferation-associated genes (namely *BUB1, CCNB2, CDK1, CDKN3, FOXM1, KIAA0101, MAD2L1, MELK, MKI67,* and *TOP2A)*, was identified as a marker of response to ICIs in NSCLC. Poorly, moderately, and highly proliferative tumors were somewhat equally represented in NSCLC, with tumors with the highest PD-L1 expression being more frequently moderately proliferative as compared to lesser levels of PD-L1 expression. Proliferation status had an impact on survival in patients with both PD-L1 positive and negative tumors. There was a significant survival advantage for moderately proliferative tumors compared to their combined highly/poorly counterparts (*p* = 0.021). Moderately proliferative PD-L1 positive tumors had a median survival of 14.6 months that was almost twice that of PD-L1 negative highly/poorly proliferative at 7.6 months (*p* = 0.028). Median survival in moderately proliferative PD-L1 negative tumors at 12.6 months was comparable to that of highly/poorly proliferative PD-L1 positive tumors at 11.5 months, but in both instances less than that of moderately proliferative PD-L1 positive tumors. Similar to survival, proliferation status has impact on disease control (DC) in patients with both PD-L1 positive and negative tumors. Patients with moderately versus those with poorly or highly proliferative tumors have a superior DC rate when combined with any classification schema used to score PD-L1 as a positive result (i.e., TPS ≥ 50% or ≥ 1%), and best displayed by a DC rate for moderately proliferative tumors of no less than 40% for any classification of PD-L1 as a negative result. While there is an over representation of moderately proliferative tumors as PD-L1 expression increases this does not account for the improved survival or higher disease control rates seen in PD-L1 negative tumors.

**Conclusions:**

Cell proliferation is potentially a new biomarker of response to ICIs in NSCLC and is applicable to PD-L1 negative tumors.

**Electronic supplementary material:**

The online version of this article (10.1186/s40425-019-0506-3) contains supplementary material, which is available to authorized users.

## Background

On March 4th 2015, nivolumab (Opdivo®, from Bristol-Myers Squibb) became the first immune checkpoint inhibitor (ICI) to be approved by the US Food and Drug Administration for use in patients with metastatic nonsquamous non-small cell lung cancer (NSCLC) progressing on or after platinum-based chemotherapy [1], following disclosure of the results from the Phase III Checkmate-037 trial [2]. Since then, three other ICIs that inhibit the programmed cell death pathway, including programmed cell death 1 (PDCD1 or CD279, best known as PD-1) and its ligands – CD274 (best known as PD-L1) and programmed cell death 1 ligand 2 (PDCD1LG2 or CD273, best known as PD-L2) – have been licensed for use in NSCLC patients, namely pembrolizumab (Keytruda®, from Merck) [3, 4], atezolizumab (Tecentriq®, from Genentech) [5, 6], and durvalumab (Imfinzi®, from AstraZeneca) [7]. Response rates to these ICIs employed as single agent immunotherapeutic interventions in an unselected population, however, is generally below 20% [3]. Moreover, ICI-based immunotherapy has been estimated to cost 100,000–250,000 USD per patient (with some variation depending on specific ICI, treatment regimen and duration) [8]. Thus, considerable efforts are being devoted to the elucidation of the mechanisms controlling the development of primary and acquired resistance to ICIs [9], as well as to the identification of biomarkers with robust predictive value [10, 11].

These observations have rapidly been translated into the clinical management of NSCLC with the FDA companion diagnostic for pembrolizumab treatment, PD-L1 expression levels assessed by the PD-L1 22C3 pharmDx assay (from Agilent) [12]. However, response prediction based on PD-L1 levels is not 100% accurate. For instance, pembrolizumab monotherapy in NSCLC patients with a PD-L1 tumor proportion score (TPS) < 1% (i.e., membranous PD-L1 expression on < 1% malignant cells), of 1–49%, and ≥ 50% was associated with response rates of 10.7, 16.5, and 45.2%, respectively [3]. Thus, a small population of NSCLC patients with low PD-L1, seemingly “negative biomarker” patients, will still respond to ICI-based therapy. Conversely, not all patients with high PD-L1 TPS obtain clinical benefits from ICIs, which suggests the existence of alternative resistance mechanisms, such as mutations that affect the ability of cancer cells to be recognized or eliminated by the immune system [9], or other mechanism of local immunosuppression in the tumor microenvironment via pathways that do not directly involve ICI targets such as PD-L1 and PD-1 [3].

We employed targeted RNA sequencing of an immune related panel of slightly less than 400 genes to optimize the detection of low expressing genes as opposed to whole transcriptome, that was specifically designed for use in formalin fixed paraffin embedded (FFPE) clinical samples [13]. This list of genes was divided into 41 different immune function categories and analyzed for response to ICIs in a cohort of NSCLC patients from ten different institutions. The highest association with response among the different immune function categories was cell proliferation, represented by the expression of ten unique genes. We demonstrate that either extreme of cellular proliferation in the tumor microenvironment, i.e. highly or poorly proliferative, is associated with resistance to ICIs amongst NSCLC patients, and that the expression levels of a 10-gene set associated with cellular proliferation can be harnessed to improve patient stratification beyond PD-L1 TPS. Most importantly, we show that additional stratification of PD-L1 negative NSCLC based upon cell proliferation status introduces a new approach to response to ICI therapy in NSCLC.

## Methods

### Patients and clinical data

**Fig. 1:**
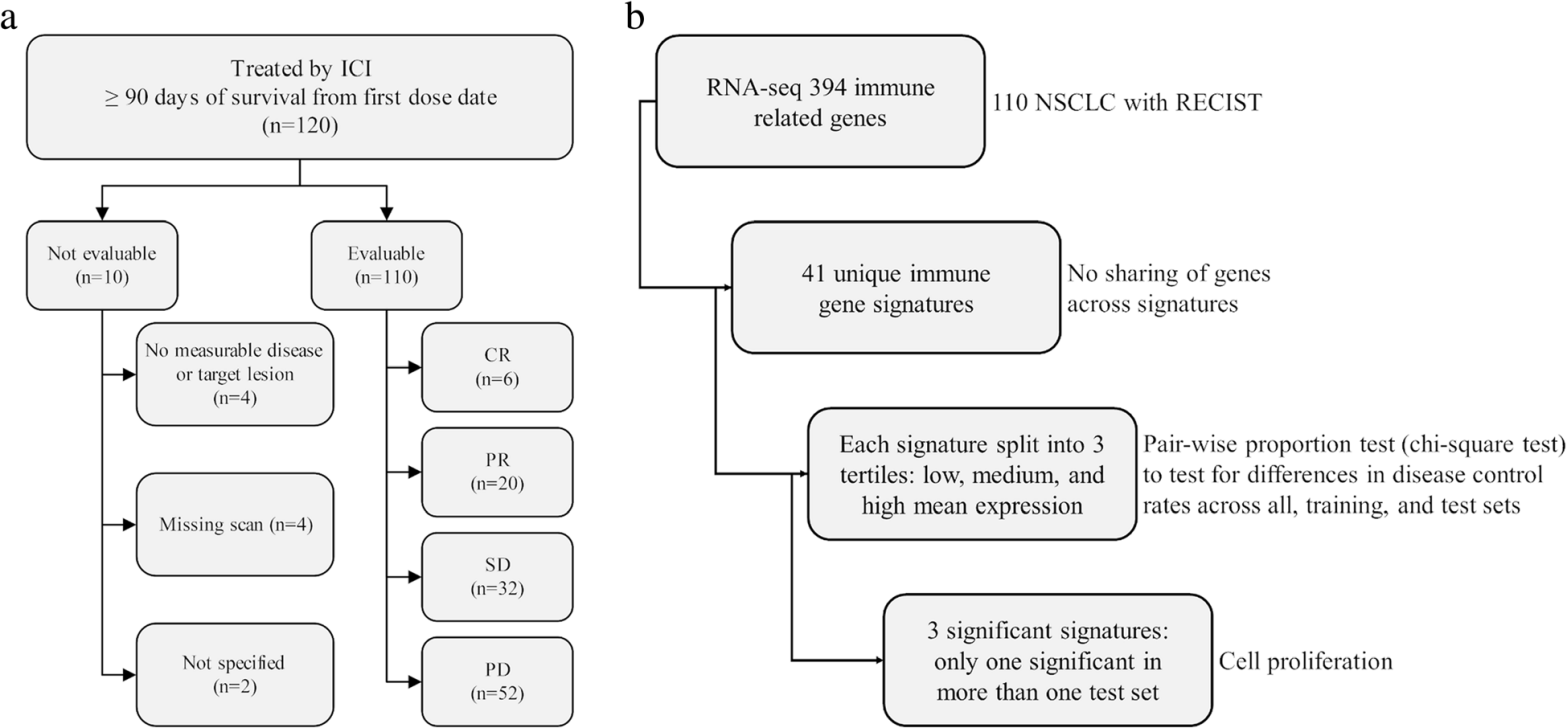
Summary of patient disposition and exploratory analysis. **a**) A total of 120 patients previously treated with checkpoint inhibitors were included in the study. All patients had survival data from date of first dose of checkpoint inhibitor, while 110 were evaluable by RECIST v1.1 for response. **b**) Exploratory analysis using pair-wise proportion test of 41 immune-related gene functions derived from 394 genes for patients with disease control versus no disease control identifies cell proliferation as a biomarker of interest

Ten collaborating institutions obtained approval by their respective institutional review boards (IRBs) to submit existing de-identified specimens and associated clinical data for use in this study. A total of 120 patients were included in the study (Fig. 1a), based on the following criteria: (1) history of Stage IV NSCLC; (2) availability of adequate archival formalin-fixed paraffin-embedded (FFPE) tissue collected prior to treatment with ICIs; (3) availability of sequencing data; and (4) availability of demographic, diagnosis, follow-up and survival data. Table 1 summarizes the baseline clinical characteristics of these patients (individual patient data provided in Additional file 1: Table S1).

**Table 1:** Patient characteristics

	Patients (*n* = 120)
Age at initial diagnosis (years)	
< 30	1 (00.0)
30–39	1 (00.0)
40–49	4 (03.3)
50–59	28 (23.3)
60–69	43 (35.8)
70–79	34 (28.3)
≥ 80	9 (07.5)
Mean	65
Sex	
Female	61 (50.8)
Male	59 (49.2)
Race	
White	96 (80.0)
Other	17 (14.2)
Unknown	7 (05.8)
Vital status at last follow up	
Alive	60 (50.0)
Dead	60 (50.0)
Checkpoint inhibitor	
atezolizumab	2 (01.7)
ipilimumab + nivolumab	2 (01.7)
nivolumab	79 (65.8)
pembrolizumab	37 (30.8)

Patients who were treated with ICIs were included if they were treated by an agent approved by the FDA as of November 2017 and had follow up and survival from first ICI dose (*n* = 120). ICI-treated patients who died within 90 days of first dose were excluded as it could not be discerned whether they were rapid progressors or had poor performance prior to going on drug. Patients lacking sufficient follow up time for response evaluation (less than 90 days from first dose), were also excluded from analysis. Of the120 ICI-treated patients, for all of which survival data was available, there were 10 patients not evaluable for response due to either no measurable disease or target lesion (*n* = 4), missing scans (*n* = 4), or not specified (*n* = 2) (Fig. 1a). For the remaining 110 patients all were evaluable for response based on RECIST v1.1 and were divided into a test set (*n* = 34) from one institution with the most patients (Duke) and a training set (*n* = 76) from all other institutions. Patients whose best response was complete response (CR), partial response (PR), or stable disease (SD) with 12 months or more survival were classified as disease control (DC), while patients whose best response was progressive disease (PD) or SD with less than 12 months survival were classified as no disease control (NDC). Duration of response was not available for all patients and not included for final analysis.

### Immunohistochemical studies

The expression of PD-L1 on the surface of cancer cells was assessed in all cases by means of the Dako Omnis Platform and the 22C3 pharmDx antibody (Agilent, Santa Clara, CA) using FDA-scoring guidelines [14]. Briefly, a minimum of 100 viable tumor cells were assessed for membranous staining of any intensity for the 22C3 antibody. The percentage of viable tumor cells showing partial or complete membrane staining relative to all viable tumor cells present in the sample (positive and negative) was then used to derive a tumor proportion score (TPS). PD-L1 levels were scored by a board-certified anatomic pathologist as per published guidelines [15], with a TPS ≥ 50% considered as a strongly positive result for different comparisons, while a result of ≥1% considered as positive result for different comparisons. PD-L1 TPS ≥ 1% to < 50% were considered weakly positive for additional comparative purposes. PD-L1 TPS < 1% was considered as negative. Ki-67 positivity amongst neoplastic and immune cells was scored upon nuclear staining, regardless of intensity, with the M7240 (clone MIB1) antibody from Dako (Carpentaria, CA) with the percentage of each cell type recorded.

### RNA-seq

RNA were extracted from each sample and processed for targeted RNA-seq, as previously described [13, 16]. Gene expression was evaluated by amplicon sequencing of 394 immune transcripts on samples that met validated quality control (QC) thresholds [13].

### Data analysis

Immune gene expression ranks (range 0–100) from a targeted RNA-seq immune panel of approximately 400 genes were divided into 41 biological function categories according to commercial annotations from the manufacturer (Additional file 1: Table S2). For all 110 cases with response, distribution of each biological function was split into 3 tertiles of low (less than 33), medium (between 33 and 66) and high (greater than 66). Next, we performed a pair wise proportion test (chi-square test) to test for difference in DC rates for these three tertiles (i.e. low vs medium, medium vs high and low vs high) for each biological function (Fig. 1b). Proportion test was performed with continuity correction and pairwise *p*-values for each biological function were adjusted for multiple hypothesis testing using “holmes” correction. We further divided the dataset into a training set (*n* = 76) consisting of samples from all data access groups except the largest contributor. A separate test set (*n* = 34) was constituted from samples from a single largest contributing institute. Any biological function that did not have cases representing one or more tertiles was removed from further analysis due to lack of dynamic range of that biological function in the population assessed in this study. The most significant gene functions were utilized for further analysis. Survival analysis was performed using a log-rank test on 5-year Kaplan-Meier survival curves for PD-L1 levels assessed by IHC and combined expression of 10 proliferation-related genes assessed by RNA-Seq. Comparison of DC rate was performed using Chi-square test with Yate’s continuity correction. Multivariate analysis was performed by fitting a binomial logistic regression model to DC labels and co-variates such as proliferation status, PD-L1 status, histology, race, sex, and age category. Analysis of variance (ANOVA) was performed on the fitted model to study the table of deviance to determine the co-variate that explains the most variance in the DC rates.

## Results

### Immune-related gene functions

Among 41 different immune-related gene functions (Additional file 1: Table S2) evaluated by pairwise comparison test in the training set (*n* = 76), three were significantly differentially expressed for DC versus NDC for at least one comparison (Additional file 1: Table S3). These three functions and specific genes (see Additional file 1: Table S2 for full gene names) included *proliferation* [*BUB1*, *CCNB2*, *CDK1*, *CDKN3*, *FOXM1*, *KIAA0101*, *MAD2L1*, *MELK*, *MKI67 (*better known as *Ki-67),* and *TOP2A*; maximum *p* = 0.0092], *antigen processing* (*CD74, HLA-A, HLA-B, HLA-C, HLA-DMA, HLA-DMB, HLA-DOA, HLA-DOB, HLA-DPA1, HLA-DPB1, HLA-DQA1, HLA-DQA2, HLA-DQB2, HLA-DRA, HLA-DRB1, HLA-E, HLA-F, HLA-F-AS1, HLA-G*; *p* = 0.0796), and *dendritic cell* (*HERC6, IL3RA, ITGAX, NRP1, TLR3, ZBTB46*; *p* = 0.0903). When both the training and test set (*n* = 110) were used for the same comparison (Additional file 1: Table S4), proliferation was the only of these three functions that was significant (Fig. 1b). Results for the test set (*n* = 34) did not identify proliferation, antigen processing, or dendritic cell as significant (Additional file 1: Table S5), presumably due to the small size of the sample set. Proliferation was chosen for further evaluation based upon the identification as a significant factor in the training set as well as the combination of the training and test set.

### Proliferative status

**Fig. 2:**
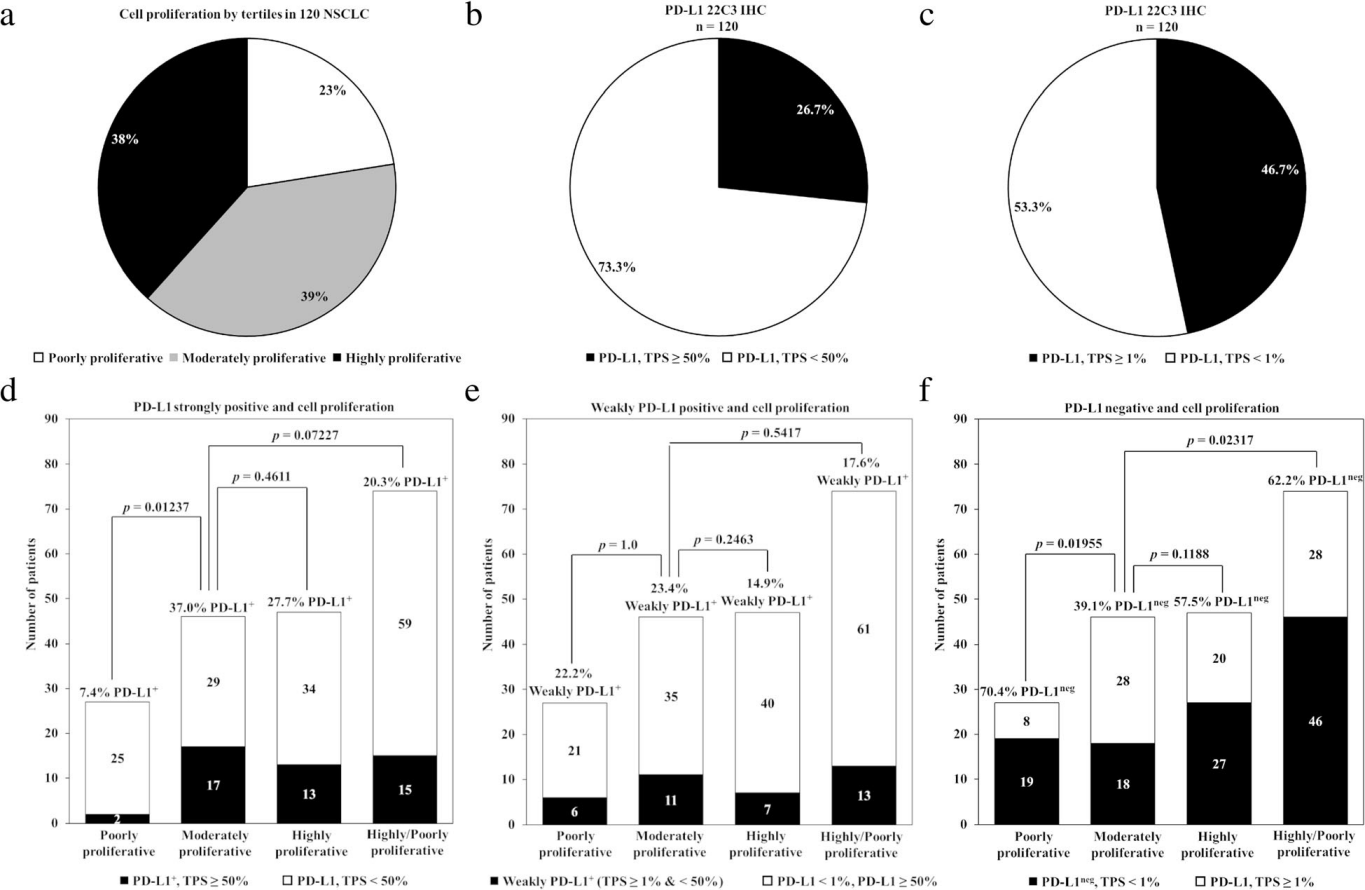
Results for cell proliferation as an independent biomarker. **a**) Proportion of 120 NSCLC patients for cell proliferation by tertiles of poorly, moderately, and highly proliferative. **b**) Proportion of 120 NSCLC patients positive or negative for PD-L1 IHC using a cut-off of tumor proportion score of ≥50% as a positive result. **c)** Proportion of 120 NSCLC patients positive or negative for PD-L1 IHC using a cut-off of tumor proportion score of ≥1% as a positive result. **d**) Prevalence for all combinations of strongly positive PD-L1 (TPS ≥ 50%) cases and proliferation status. **e**) Prevalence for all combinations of PD-L1 and proliferation status for weakly positive PD-L1 cases (TPS ≥ 1 and < 50%). **f**) Prevalence for all PD-L1 negative (TPS < 1%) cases and proliferation status. Number and *p* values are reported

NSCLC had a wide distribution of poorly, moderately, and highly proliferative tumors with input by both neoplastic and immune cells that can be measured in more than one way. The mean expression rank values of 10 proliferation-related genes in 120 NSCLC specimens (adenocarcinoma *n* = 94, sarcomatoid carcinoma *n* = 1, squamous cell carcinoma *n* = 25) was used as the primary indicator for the proliferative status of the tumor microenvironment. Tumors were stratified into poorly, moderately and highly proliferative based on the tertile rank of expression of this gene signature as compared to a separate reference population of 167 patients with multiple tumor types (Additional file 1: Table S6) [10]. Based on this analysis, poorly proliferative tumors were the least frequent in all available samples tested (27/120; 22.5%), followed by an equal distribution of highly (47/120; 39.2%) and moderately proliferative tumors (46/120; 38.3%), (Fig. 2a).

**Fig. 3:**
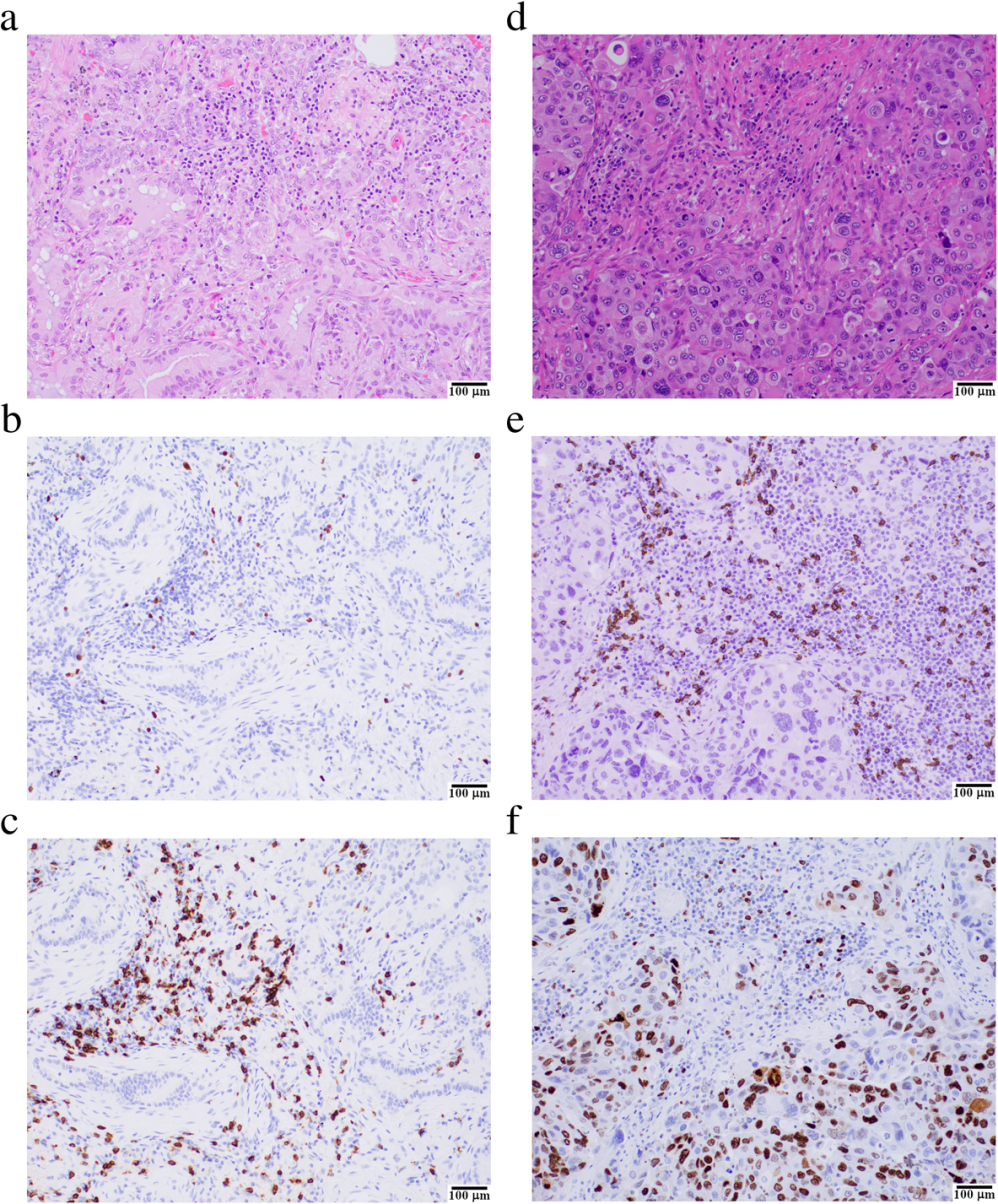
Immunohistochemical assessment of Ki-67 positivity and CD8^+^ T cell infiltration. Representative fields for hematoxylin/eosin (**a, d**), CD8 positivity (**b, e**) and Ki-67 positivity (**c, f**) are depicted. The left hand panel (**a-c**) of a poorly proliferative tumor shows numerous CD8+ T-cells (**c**), while Ki-67 (**b**) stains very few neoplastic or immune cells. The right hand panel (**d-f**) of a highly proliferative tumor like the other case shows numerous CD8^+^ T-cells (**f**), while Ki-67 (**e**) stains a high number of neoplastic and immune cells. Scale bar = 100 μm

To define whether neoplastic cells, immune cells, or both constituted the source of proliferation-related transcripts, 7 highly proliferative and 9 poorly proliferative cases were evaluated by immunohistochemistry for the expression of MKI67 (best known as Ki-67), a biomarker of proliferation largely employed in the clinics [17]. Highly proliferative tumors (as defined by RNA-seq) had > 50% of neoplastic cells staining positive for Ki-67 in 6 out of 7 cases, while their poorly proliferative counterparts contained less than 40% Ki-67^+^ neoplastic cells in 8 of 9 cases (Additional file 1: Table S7). In a similar fashion, highly proliferative tumors had 5% or more of immune cells staining positive for Ki-67 in all cases, while their poorly proliferative counterparts showed only two cases with this degree of reactivity. Importantly, an abundant tumor CD8^+^ T-cell infiltrate did not necessarily correlate with a highly proliferative tumor microenvironment. For example, in one poorly proliferative adenocarcinoma (Fig. 3a) there is a lack of staining by Ki-67 in both malignant and immune cells (Fig. 3b), even though there is an abundance of CD8^+^ T cells (Fig. 3c). In comparison, for a highly proliferative adenocarcinoma (Fig. 3d) there is frequent staining by Ki-67 in both malignant and immune cells (Fig. 3e), with a similar number of CD8^+^ T cells (Fig. 3f).

To evaluate the impact of single gene proliferation results, e.g. Ki-67, the mean expression rank values of all 10 proliferation-related genes were evaluated for accuracy (i.e. true positive plus true negatives divided by total number of results) for each gene individually (Additional file 1: Table S6). Accuracy ranged from a low of 52.7% for FOXM1 to a high of 67.3% for TOP2A, as compared to a value of 71.8% for the mean expression rank values of all ten proliferation-related genes (Additional file 2: Figure S1). The accuracy of Ki-67 at 59.1% was near the mid-value of other single gene results.

The sum of all of these results suggest that poorly, moderately, and highly proliferative tumors are somewhat equally represented in NSCLC; that both immune cells and malignant cells are sources of proliferation-related transcripts, and it is possible to reach similar results for any of the 10 genes using only single gene evaluations.

### PD-L1 expression

Tumors with the highest PD-L1 expression were more frequently moderately proliferative as compared to lower levels of PD-L1 expression. PD-L1 TPS, defined as the percentage of neoplastic cells displaying membranous positivity of any intensity upon staining with the DAKO 22C3 antibody, ranged from 0 to 100 and 32/120 (26.7%) of all cases were strongly positive (Fig. 2b), while 56/120 (46.7%) of all cases were positive at any level of staining (Fig. 2c). Moderately proliferative tumors were slightly enriched for strongly positive PD-L1 tumors as compared to highly proliferative tumors (*p =* 0.4611), and more so as compared to poorly proliferative tumors *(p =* 0.01237), or a combination of the latter two (*p* = 0.07227), (Fig. 2d). For weakly positive PD-L1 tumors, moderately proliferative were not enriched as compared to poorly proliferative counterparts (*p =* 1.0), highly proliferative (*p =* 0.2463), or a combination of the latter two (*p* = 0.5417), (Fig. 2e). For PD-L1 negative tumors, moderately proliferative were under represented as compared to poorly proliferative counterparts (*p =* 0.01955), or a combination of poorly and highly proliferative (*p* = 0.02317), but less so for highly proliferative (*p =* 0.1188), (Fig. 2f). Overall these results support that as PD-L1 expression increases there is an over representation of moderately proliferative tumors, but as shown below does not account for the improved survival or higher disease control rates seen in PD-L1 negative tumors.

### Overall survival

**Fig. 4:**
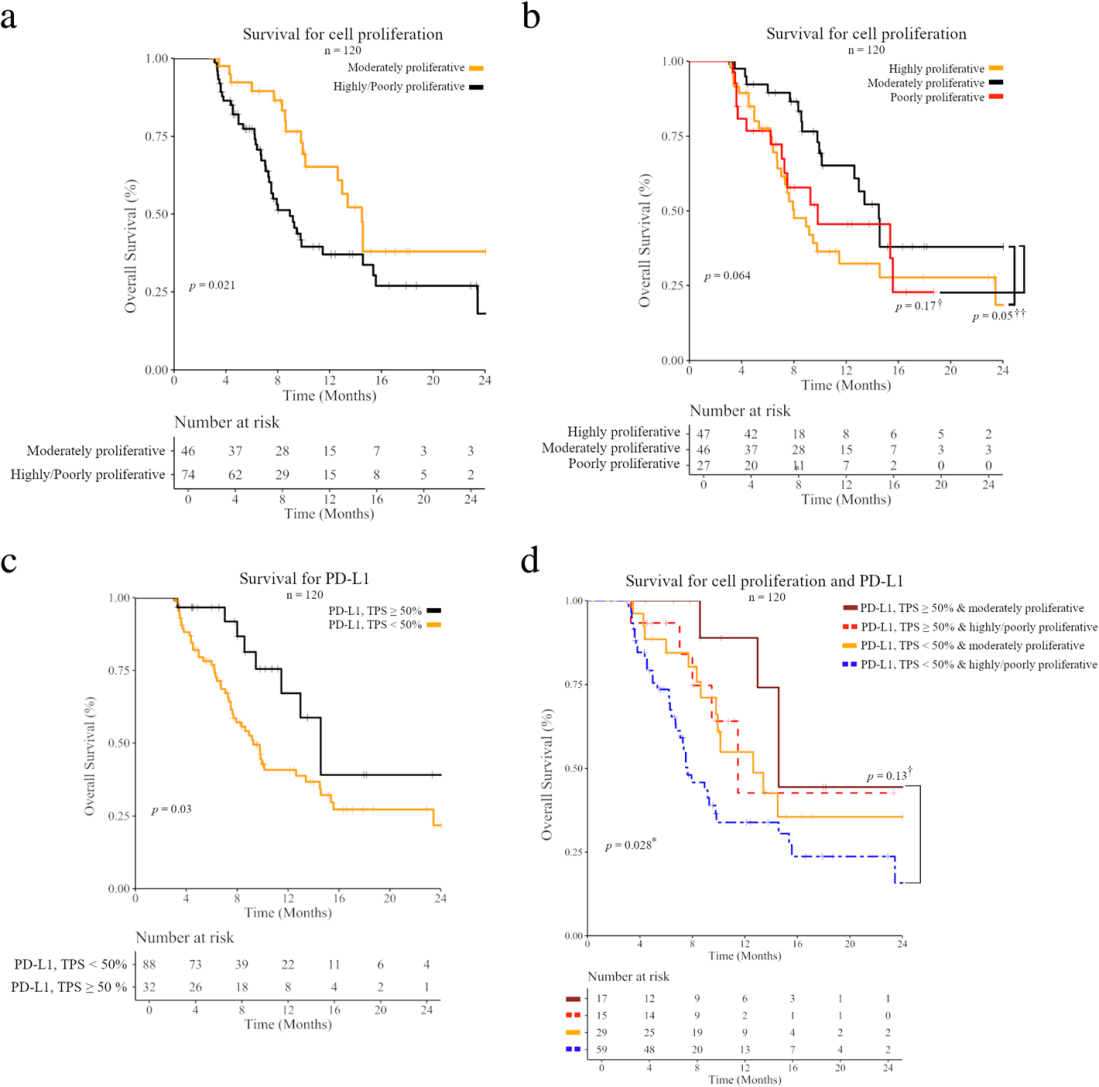
Overall survival of 120 NSCLC patients receiving an immune checkpoint inhibitor (ICI) as part of their therapy. **a**) Overall survival based upon stratification by cell proliferation for moderately versus combined poorly/highly proliferative. **b**) Overall survival based upon stratification by cell proliferation for moderately versus poorly and highly proliferative. **c**) Overall survival based upon stratification by PD-L1 expression levels using TPS ≥ 50% as a cut-off for a positive result. **d**) Overall survival based upon stratification by strongly positive PD-L1 tumors and proliferation status (PD-L1 TPS ≥ 50% moderately proliferative, PD-L1 TPS ≥ 50% highly or poorly proliferative, PD-L1 TPS ≥ 50% moderately proliferative, PD-L1 TPS ≥ 50% highly or poorly proliferative). Number at risk and *p*-values are reported

Proliferation status had an impact on survival in patients with both PD-L1 positive and negative tumors. There was a significant survival advantage for moderately proliferative tumors compared to their combined highly/poorly counterparts (*p* = 0.021) (Fig. 4a). When highly and poorly proliferative groups were evaluated separately there was a trend towards survival for patients with moderately proliferative tumors (*p* = 0.064) (Fig. 4b). Likewise, the survival of patients with strongly positive PD-L1 tumors was associated with a statistically significant survival advantage (*p* = 0.03) (Fig. 4c). A combination of proliferation and PD-L1 resulted in a significant survival advantage in moderately proliferative strongly positive PD-L1 tumors with a median survival of 14.6 months that was almost twice that of all less than strongly positive PD-L1 highly/poorly proliferative tumors at 7.6 months (*p* = 0.028) (Fig. 4d). Likewise, median survival in less than strongly positive PD-L1 moderately proliferative tumors at 12.6 months was comparable to that of highly/poorly proliferative strongly positive PD-L1 tumors at 11.5 months (*p* = 0.86) (Fig. 4d), but in both instances less than that of moderately proliferative strongly positive PD-L1 tumors. The results for all PD-L1 positive tumors by a TPS ≥ 1% criteria were very similar (Additional file 3: Figure S2). The summary of these results support that moderately proliferative tumors have a survival advantage beyond PD-L1 positive tumors for NSCLC patients treated with checkpoint inhibitors.

### Disease control rate

**Fig. 5:**
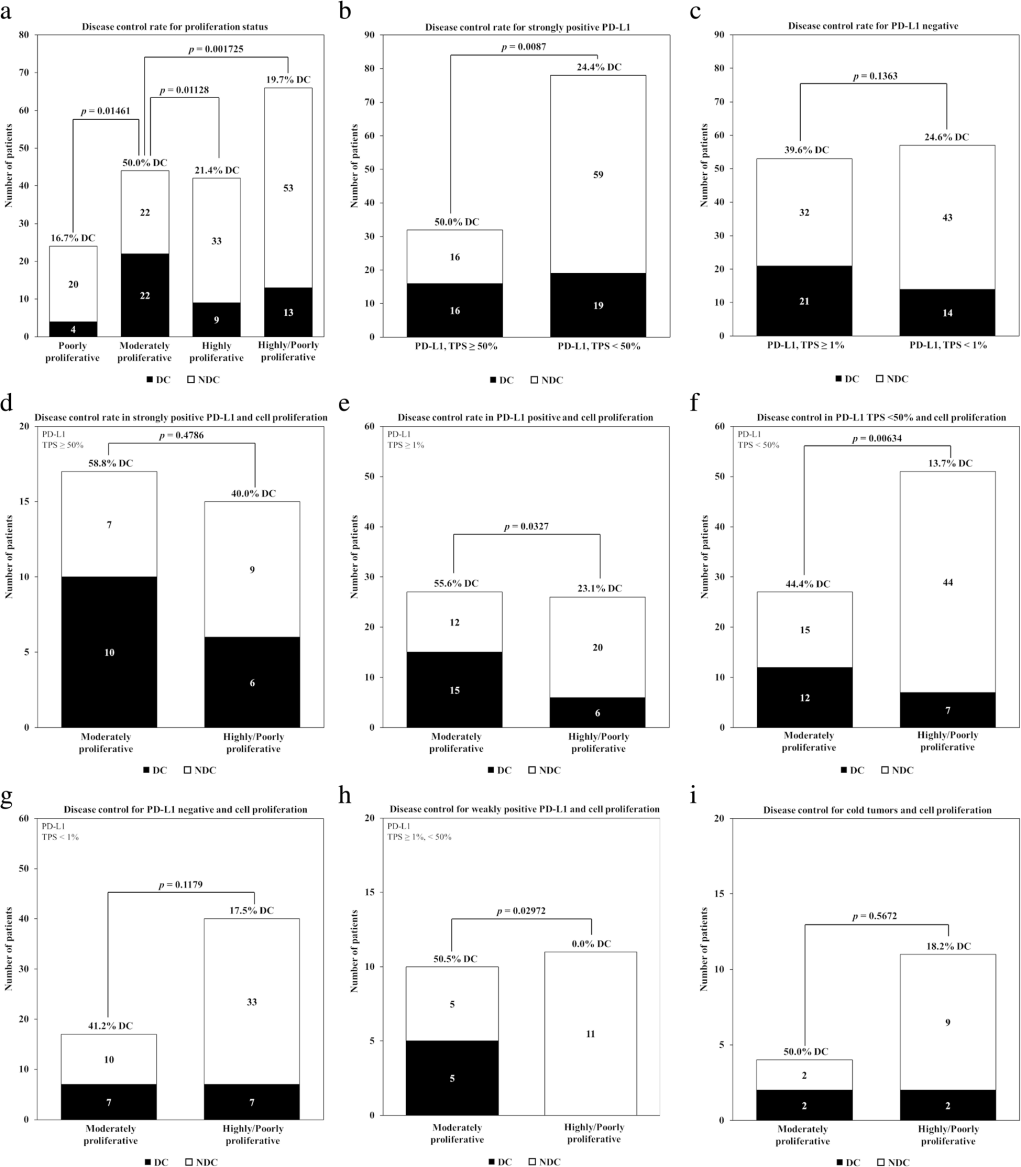
Impact of PD-L1 levels and proliferative status on disease control rate in 110 NSCLC patients receiving an immune checkpoint inhibitor (ICI) as part of their therapy. **a**) Prevalence and DC rate for moderately versus highly and poorly proliferative tumors, as well as combined of the latter two. **b**) Prevalence and DC rate for strongly positive PD-L1 (TPS ≥ 50%). **c**) Prevalence and DC rate for PD-L1 negative (TPS < 1%). **d**) Prevalence and DC rate for strongly positive PD-L1 combined with moderately versus highly/poorly proliferative tumors. **e**) Prevalence and DC rate for PD-L1 positive (TPS ≥ 1%) combined with moderately versus highly/poorly proliferative tumors. **f**) Prevalence and DC rate for PD-L1 less than strongly positive (TPS < 50%) combined with moderately versus highly/poorly proliferative tumors. **g**) Prevalence and DC rate for PD-L1 negative (TPS < 1%) combined with moderately versus highly/poorly proliferative tumors. **h**) Prevalence and DC rate for weakly positive PD-L1 (TPS ≥ 1% and < 50%) combined with moderately versus highly/poorly proliferative tumors. **i**) Prevalence and DC rate for minimal tumor infiltration by CD8^+^ T cells (so-called “cold” tumors) combined with moderately versus highly/poorly proliferative tumors

Similar to survival, proliferation status had an impact on disease control in patients with both PD-L1 positive and negative tumors. The overall objective of evaluating disease control was to show this intersection of response to checkpoint inhibition for cell proliferation versus the current standard of PD-L1 IHC. The results (Table 2, Fig. 5) show that patients with moderately versus those with poorly or highly proliferative tumors have a superior DC rate when combined with any classification schema used to score PD-L1 as a positive result (i.e., TPS ≥ 50% or ≥ 1%; see Additional file 4 for full results). The value of cell proliferation as a marker of response was best displayed by noting that the DC rate for moderately proliferative tumors was no less than 40% for any classification of PD-L1 as a negative result. This was critically important for the fifty-seven negative PD-L1 negative tumors for which moderately proliferative tumors had a DC rate of 41.2% (7/17) (Fig. 5g), while the DC rate among highly and poorly proliferative tumors combined was 17.5% (7/40, *p* = 0.1179). The summary of all of these results support that cell proliferation is a relevant biomarker in all groups of NSCLC, but is unique and clinically useful for patients with PD-L1 negative tumors. Further support of this conclusion was a multivariate analysis on all co-variates using binomial logistic regression model showed that moderately proliferative tumors to have a significant association with probability of disease control (Table 3; *p* = 0.0071). Furthermore, analysis of deviance of each co-variate (Table 3) suggests that adding proliferation to a null model improved it significantly (*p* = 0.0009) followed by a second most informative co-variate of PD-L1 status (*p* = 0.0337). Collectively these results suggest that, the proliferative status of the tumor microenvironment can be harnessed to improve patient stratification based on PD-L1 expression levels. Importantly, cell proliferation seems to have value as a biomarker of response in PD-L1 negative tumors.

**Table 2:** Disease control for cell proliferation and PD-L1 IHC

Cell Proliferation	PD-L1 IHC	DC	NDC	Total pts	DC rate	*χ2 test*
Moderately		22	22	44	50.0%	
Highly		9	33	42	21.4%	*p* = 0.0146
Poorly		4	20	24	16.7%	*p* = 0.0113
Poorly/highly		13	53	66	19.7%	*p* = 0.0017
	Strongly positive (TPS ≥ 50%)	16	16	32	50.0%	
	Not strongly positive (TPS < 50%)	19	59	78	24.4%	*p* = 0.0009
	Positive (TPS ≥ 1%)	21	32	53	39.6%	
	Negative (TPS < 1%)	14	43	57	24.6%	*p* = 0.1363
Moderate	Strongly positive (TPS ≥ 50%)	10	7	17	58.8%	
Poorly/highly		6	9	15	40.0%	*p* = 0.4786
Moderately	Not strongly positive (TPS < 50%)	12	15	27	44.4%	
Highly		4	25	29	13.8%	*p* = 0.0250
Poorly		3	19	22	13.6%	*p* = 0.0438
Poorly/highly		7	44	51	13.7%	*p* = 0.0063
Moderately cold tumors (CD8 rank < 15%)		7	10	17	41.2%	
Poorly/highly cold tumors (CD8 rank < 15%)		7	33	40	17.5%	*p* = 0.1179
Moderately cold tumors (CD8 rank < 33%)		5	5	10	50.0%	
Poorly/highly cold tumors (CD8 rank < 33%)		0	11	11	0.0%	*p* = 0.3298

**Table 3:** Multivariate analysis

Variable		Estimate	Std. Error	*z* value	*p* value
(Intercept)		17.3526	2712.1561	0.006	0.9949
Proliferation Moderately		1.3503	0.5013	2.694	0.00707
PD.L1. status Positive		0.5169	0.547	0.945	0.34468
Histology SCC.or.Other		−0.5898	0.6417	−0.919	0.35801
race Black or African American		−34.8319	3301.0002	−0.011	0.99158
race Black or African American		−16.7776	2712.156	−0.006	0.99506
race Other		−35.6219	4796.5772	−0.007	0.99407
race Other		−34.6736	4796.5771	−0.007	0.99423
race Unknown		−18.5693	2712.1561	−0.007	0.99454
race White		−17.9126	2712.1559	−0.007	0.99473
race White		−18.3781	2712.1559	−0.007	0.99459
sex M		0.1522	0.5119	0.297	0.76616
age_cat 1–29		−35.0709	4796.5772	−0.007	0.99417
age_cat 40–49		−1.201	1.6759	−0.717	0.47359
age_cat 50–59		−0.6471	0.9129	−0.709	0.47843
age_cat 60–69		−0.9142	0.8863	−1.031	0.30233
age_cat 70–79		−1.1416	0.9098	−1.255	0.20955
Analysis of deviance of each co-variate					
Co-variate	Df	Deviance	Resid. Df	Resid. Dev	*P* value(>Chi)
NULL			109	137.61	
Proliferation	1	11.1163	108	126.49	0.0008557
PD.L1.status	1	4.5112	107	121.98	0.0336733
Histology	1	0.0593	106	121.92	0.8076295
race	7	7.4867	99	114.44	0.3800195
sex	1	0.1064	98	114.33	0.7442778
age_cat	5	4.2582	93	110.07	0.5128654

### Proliferative status and cold tumors

Proliferation status had an impact on disease control in patients with factors other than PD-L1 positive or negative status, impacting response to checkpoint inhibitors. In this regard, cell proliferation was further evaluated for value beyond PD-L1 status in the emerging recognition of inflammatory status [16], and more specifically the degree of CD8 infiltration. Response was evaluated for tumors with reduced levels of CD8-coding transcripts as compared to a reference population of 167 patients with multiple tumor types, which we previously demonstrated to indicate minimal tumor infiltration by CD8^+^ T cells (so-called “cold” tumors) [16]. As there is no current absolute criteria to define cold tumors we first arbitrarily defined this group by a CD8 rank less than 15, and then compared to those results to an non-arbitrary cut-off of the lower tertile of CD8 rank, or a value less than 33. Irrespective of the cut-off, DC was accurately predicted by the proliferative status of the tumor microenvironment (Table 2), although the numbers are quite small for the more stringent cut-off value (Fig. 5i). Most importantly, the DC rate was greater than 50% for any grouping of moderately proliferative cold tumors, while the rate was less than 20% for poorly/highly proliferative counterparts. PD-L1 status did not associate with response in cold tumors (Additional file 1: Table S8), again supporting that cell proliferation is a unique biomarker of response in NSCLC.

## Discussion

Our findings suggest that a highly or poorly proliferative tumor microenvironment is associated with limited sensitivity to ICIs amongst NSCLC patients, and that targeted RNA-seq can be employed to assess the proliferative status of the tumor microenvironment at diagnosis, with the ultimate goal of improving clinical decision making based on PD-L1 only. Most importantly, these findings suggest that some highly or poorly proliferative tumors may be resistant to ICIs independent of PD-L1 or inflamed status and that both PD-L1 positive and PD-L1 negative tumors at any TPS value can be stratified more accurately by cell proliferation. Moving forward the need for standardization of cell proliferation will be vitally important in comparing response among various studies. In that regard the proliferative potential of malignant cells (assessed by Ki-67 positivity or enumeration of mitotic figures) has been extensively employed over the past 3 decades for prognostic purposes in a number of tumors [18–21]. In our study, Ki-67 as measured by RNA-seq analysis was not the most accurate predictor of disease control as a single gene result, but rather was TOP2A. At such a formative stage of development we did not evaluate proliferation as a continuous variable for any single gene or the mean rank of 10 genes, but this is factor that will need to evaluated further in future studies. We also did not evaluate K-67 or TOP2A IHC as a predictor of disease control and is another potential future study.

In a recent study, RNA-seq was employed to investigate the effect of proliferation on the survival of 6581 patients with 19 different cancers, as catalogued by The Cancer Genome Atlas (TCGA) [22]. In this setting, a low proliferation index was associated with improved patient survival in 7 of 19 malignancies (including lung adenocarcinoma) which were subsequently defined as “proliferation-informative cancers” [22]. Most recently, another TCGA study evaluating the immune landscape of cancer in more than 10,000 tumors identified six immune subtypes hypothesized to define immune response patterns impacting prognosis [23]. Two of these six subtypes, C1 and C2, were noted for a high proliferation rate, with both having a substantial immune component but the least favorable outcomes. In this study tumor types over represented by C1 and C2 subtypes included bladder cancer, breast cancer, cervical cancer, colon cancer, head and neck squamous cell carcinoma, lung squamous cell carcinoma, mesothelioma, ovarian cancer, gastric adenocarcinoma, and endometrial cancer. Moreover, in NSCLC, a dormant tumor-infiltrating lymphocytes (TIL) signature characterized by low activation (Granzyme B) and proliferation markers (Ki-67) in CD3 + TILs was also recently demonstrated to be associated with survival benefit in patients treated with ICI [24]. These studies support that cell proliferation should be evaluated further as an integral component of the immune response to ICIs and that results may be tumor type dependent.

While our work was not based upon a single, well-structured clinical trial, samples were obtained from 10 different institutions across the US and Europe, and results stood the test of such a heterogeneous, real-world clinical scenario. One of the major limitations of the present study is that response data (based on RECIST v1.1) was available for a relatively small number of cases (110 patients), which obliged us to operate on pooled data from patients receiving PD-1- or PD-L1-targeting agents (nivolumab, pembrolizumab atezolizumab), CTLA4-targeting agents (ipilimumab), or both (nivolumab + ipilimumab) as it complicated subgroup analysis. As a retrospective study across multiple institutions, there were also limitations for data collection. Smoking status was not available from all sites and as such was not a variable in the multi-variate analysis. The exclusion of ICI-treated patients who died in less than 90 days post first dose checkpoint inhibitor did not allow for an analysis of this important group due to the lack of collection ECOG performance score and our subsequent inability to distinguish rapid progressors from poor health performance.

## Conclusion

In summary, we demonstrated that a poorly or highly proliferative potential in the tumor microenvironment is associated with resistance to ICI-based immunotherapy amongst NSCLC patients, and that assessing the expression levels of ten proliferation-related genes by RNA-seq in diagnostic biopsies stands out as a promising strategy for improving clinical decision making based on PD-L1 expression only. Additional studies are ongoing to test these observations in other tumor types commonly treated with ICIs.

## Additional files

Additional file 1: **Table S1.** Clinical characteristics. **Table S2.** RNA-seq gene function list. **Table S3.** Gene function analysis training set (Proportion test). **Table S4.** Gene function analysis training and test set combined (Proportion test). **Table S5.** Gene function analysis test set (Proportion test). **Table S6.** Accuracy for 10 proliferation immune-related genes. **Table S7.** Immunohistochemical assessment of Ki-67 positivity. **Table S8.** Disease control rate in cold tumors by proliferation status. (XLSX 82 kb)

Additional file 2: **Figure S1.** Gene specific proliferation values. (TIFF 274 kb)

Additional file 3: **Figure S2.** Disease control rates for PD-L1 positive (TPS > 1%) and negative tumors combined with cell proliferation status. (TIFF 345 kb)

Additional file 4: Supplementary tables with clinical annotations and data analysis results. (DOCX 32 kb)

## Abbreviations

CR: Complete Response
DC: Disease Control
FFPE: Formalin-Fixed Paraffin-Embedded
ICI: Immune Checkpoint Inhibitor
IHC: Immunohistochemistry
IRB: Institutional Review Board
NDC: No Disease Control
ORR: Objective Response Rate
OS: Overall Survival
PD: Progressive Disease
PR: Partial Response
QC: Quality Control
RECIST: Response Evaluation Criteria In Solid Tumors
SD: Stable Disease
TCGA: The Cancer Genome Atlas
TPS: Tumor Proportion Score

## References

[1] Vanpouille-Box C, Lhuillier C, Bezu L, Aranda F, Yamazaki T, Kepp O (2017). Trial watch: immune checkpoint blockers for cancer therapy. Oncoimmunology.

[2] Borghaei H, Paz-Ares L, Horn L, Spigel DR, Steins M, Ready NE (2015). Nivolumab versus docetaxel in advanced nonsquamous non–small-cell lung Cancer. N Engl J Med.

[3] Garon EB, Rizvi NA, Hui R, Leighl N, Balmanoukian AS, Eder JP (2015). Pembrolizumab for the treatment of non–small-cell lung Cancer. N Engl J Med.

[4] Merck. Keytruda (pembrolizumab) [package insert]. Whitehouse Station, NJ: U.S. Food and Drug Administration; 2018. https://www.accessdata.fda.gov/scripts/cder/daf/index.cfm?event=overview.process&ApplNo=125514.

[5] Genentech. Tecentriq (atezolizumab) [package insert]. South San Francisco, CA: U.S. Food and Drug Administration; 2018. https://www.accessdata.fda.gov/scripts/cder/daf/index.cfm?event=overview.process&ApplNo=761034.

[6] Fehrenbacher L, Spira A, Ballinger M, Kowanetz M, Vansteenkiste J, Mazieres J (2016). Atezolizumab versus docetaxel for patients with previously treated non-small-cell lung cancer (POPLAR): a multicentre, open-label, phase 2 randomised controlled trial. Lancet.

[7] AstraZeneca. Imfinzi (durvalumab) [package insert] (2018). Cambridge.

[8] Andrews A. Treating with Checkpoint Inhibitors-Figure $1 Million per Patient. Am Heal Drug Benefits. 2015;8 Spec Issue:9. http://www.ncbi.nlm.nih.gov/pubmed/26380599%5Cnhttp://www.pubmedcentral.nih.gov/articlerender.fcgi?artid=PMC4570079. PMC4570079 26380599

[9] Galluzzi L, Chan TA, Kroemer G, Wolchok JD, Lopez-Soto A. The hallmarks of successful anticancer immunotherapy. Sci Transl Med. 2018; in press. 10.1126/scitranslmed.aat7807 30232229

[10] Morrison C, Pabla S, Conroy JM, Nesline MK, Glenn ST, Dressman D (2018). Predicting response to checkpoint inhibitors in melanoma beyond PD-L1 and mutational burden. J Immunother Cancer.

[11] Nishino M, Ramaiya NH, Hatabu H, Hodi FS (2017). Monitoring immune-checkpoint blockade: response evaluation and biomarker development. Nat Rev Clin Oncol.

[12] Sul J, Blumenthal GM, Jiang X, He K, Keegan P, Pazdur R (2016). FDA approval summary: Pembrolizumab for the treatment of patients with metastatic non-small cell lung Cancer whose tumors express programmed death-ligand 1. Oncologist.

[13] Conroy JM, Pabla S, Glenn ST, Burgher B, Nesline M, Papanicolau-Sengos A (2018). Analytical validation of a next-generation sequencing assay to monitor immune responses in solid tumors. J Mol Diagnostics.

[14] Dako. PD-L1 IHC 22C3 pharmDx: Non-Small Cell Lung Cancer [interpretation manual]. Santa Clara, CA: Dako; 2015. https://www.accessdata.fda.gov/cdrh_docs/pdf15/P150013c.pdf.

[15] Büttner R, Gosney JR, Skov BG, Adam J, Motoi N, Bloom KJ (2017). Programmed death-ligand 1 immunohistochemistry testing: a review of analytical assays and clinical implementation in non–small-cell lung Cancer. J Clin Oncol.

[16] Paluch BE, Glenn ST, Conroy JM, Papanicolau-Sengos A, Bshara W, Omilian AR (2017). Robust detection of immune transcripts in FFPE samples using targeted RNA sequencing. Oncotarget.

[17] Kriegsmann M, Warth A (2016). What is better/reliable, mitosis counting or Ki67/MIB1 staining?. Transl Lung Cancer Res.

[18] Shi W, Hu J, Zhu S, Shen X, Zhang X, Yang C (2015). Expression of MTA2 and Ki-67 in hepatocellular carcinoma and their correlation with prognosis. Int J Clin Exp Pathol.

[19] Pan H, Gray R, Braybrooke J, Davies C, Taylor C, McGale P (2017). 20-year risks of breast-Cancer recurrence after stopping endocrine therapy at 5 years. N Engl J Med.

[20] Briest F, Wang Y, Arsenic R, Elezkurtaj S, Berg E, Greshake S (2018). Immunohistochemical study of mitosis-regulatory proteins in Gastroenteropancreatic neuroendocrine neoplasms. Anticancer Res.

[21] Jakobsen JN, Sørensen JB (2013). Clinical impact of ki-67 labeling index in non-small cell lung cancer. Lung Cancer.

[22] Ramaker RC, Lasseigne BN, Hardigan AA, Palacio L, Gunther DS, Myers RM (2017). RNA sequencing-based cell proliferation analysis across 19 cancers identifies a subset of proliferation-informative cancers with a common survival signature. Oncotarget.

[23] Thorsson V, Gibbs DL, Brown SD, Wolf D, Bortone DS, Ou Yang T-H (2018). The immune landscape of Cancer. Immunity.

[24] Gettinger SN, Choi J, Mani N, Sanmamed MF, Datar I, Sowell R (2018). A dormant TIL phenotype defines non-small cell lung carcinomas sensitive to immune checkpoint blockers. Nat Commun.

