# Supplementary figures and images for: Proliferative potential and resistance to immune checkpoint blockade in lung cancer patients
Source: J Immunother Cancer. 2019 Feb 1;7:27. doi: 10.1186/s40425-019-0506-3 (PMC6359802; doi:10.1186/s40425-019-0506-3)

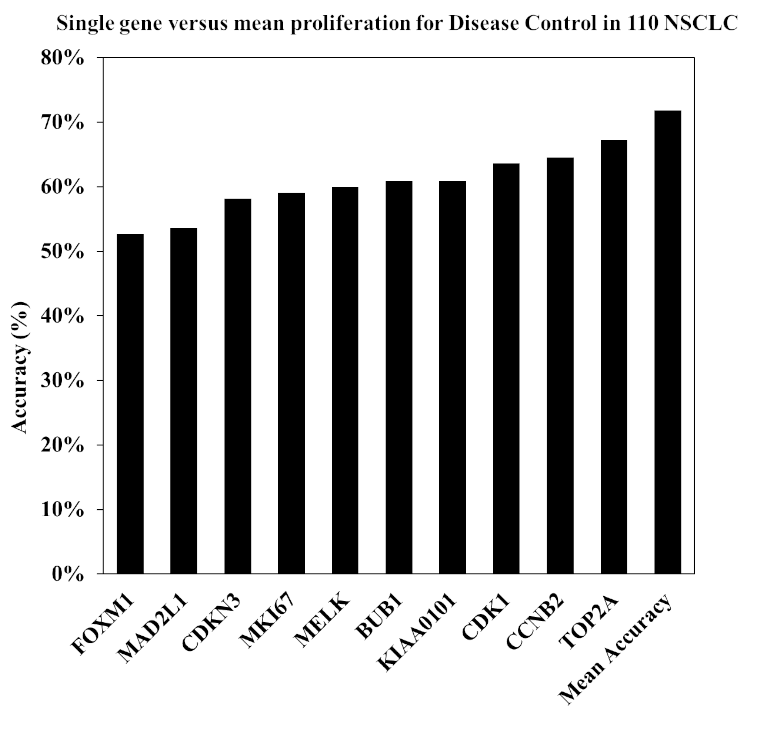

Supplement: Supplementary file 2 — Figure S1. Gene specific proliferation values. (TIFF 274 kb) [file 40425_2019_506_MOESM2_ESM.tiff]

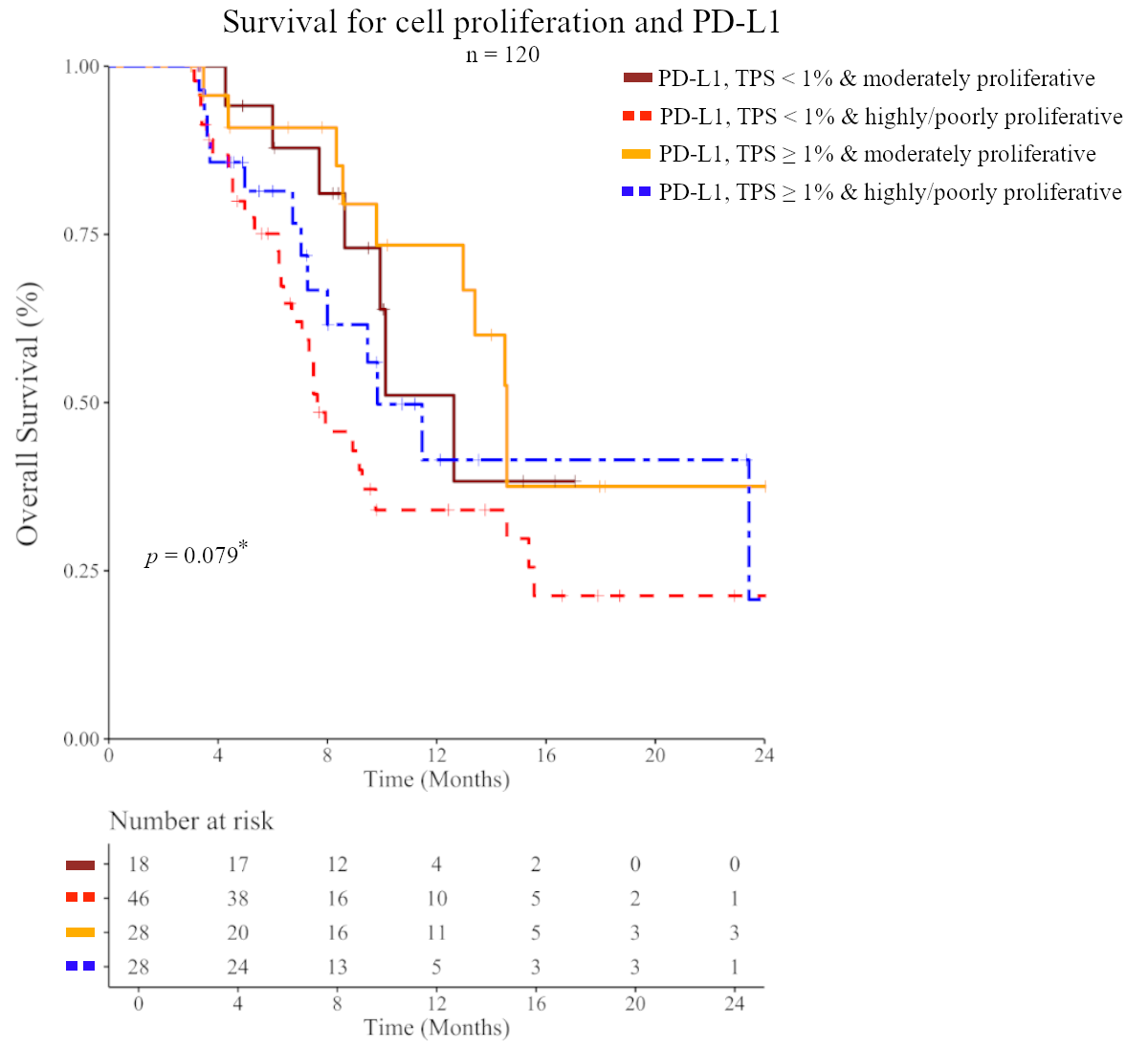

Supplement: Supplementary file 4 — Supplementary tables with clinical annotations and data analysis results. (DOCX 32 kb) [file 40425_2019_506_MOESM4_ESM.tiff]
