# Supplementary material for: Proliferative potential and resistance to immune checkpoint blockade in lung cancer patients
Source: J Immunother Cancer. 2019 Feb 1;7:27. doi: 10.1186/s40425-019-0506-3 (PMC6359802; doi:10.1186/s40425-019-0506-3)
Supplement: Supplementary file 3 — Figure S2. Disease control rates for PD-L1 positive (TPS > 1%) and negative tumors combined with cell proliferation status. (TIFF 345 kb) [file 40425_2019_506_MOESM3_ESM.docx]

**Supplementary Material - Table of Contents**

**1) Supplementary Methods** p. 02

**2) Supplementary Results** p. 05

**3) Supplementary Figures – Uploaded Separately**

**4) Supplementary Tables – See Supplementary Table Excel Document**

**5) References** p. 06

**Supplementary Methods**

**Patient characteristics, clinical data and derived variables.** All ten collaborating sites submitted complete basic patient characteristics and treatment information, including demographics (age at diagnosis, sex, race), vital status at date of last follow-up (dead/alive), and time from specimen collection to date of last follow-up or death. For ICI treated patients, name of drug, time on drug, time from specimen collection to first and last dose, and time from first dose to date of last-follow up or death were also provided. All specimens from treated patients were collected prior to first dose of checkpoint blockade therapy. All participating sites also performed retrospective chart and imaging review to either provide objective response for patients with measurable disease at baseline, or determine when patients were not evaluable, following RECIST v1.1 [1]. Qualifying baseline scans must have been performed no more than 45 days prior to first dose, and qualifying follow up scans must have been performed a minimum of 60 days post-first dose.

The variables listed below were derived from the provided data for analysis (**Table S1**) in the following manner:

**Patient class:** dichotomous variable indicating whether the submitted specimen was collected prior to (pre) or after (post) initial approval of pembrolizumab in October 2015.

**Response:** dichotomous variable indicating whether patient was a complete responder (CR), partial responder (PR) or had stable disease (SD) with a minimum of 12 months of survival time from date of first dose (Disease Control) or if they had progressive disease (PD) or stable disease (SD) with less than 12 months of survival from date of first dose (No Disease Control).

**Survival from specimen collection date:** for all patients, the number of months from date of specimen collection (surgical event) to last date of follow up or date of death.

**Survival from ICI-treated date:** for treated patients, the number of months from date of first dose of checkpoint inhibitor to last date of follow up or date of death.

**Biospecimens and pathology review.** A board-certified anatomical pathologist (CM or APS) reviewed a hematoxylin and eosin (H&E)-stained tumor section to identify the region(s) to be tested. Tumor surface area on the H&E-stained section was ≥ 2mm^2^ per slide with tumor material presenting < 50% necrosis, at least 10% or 20% neoplastic nuclei. Regions identified by the pathologist were used as guides to scrape tissue from 3-5 unstained slides, each with one tissue section of 5µm thickness affixed. Total RNA was extracted by means of the Covaris truXTRAC FFPE RNA extraction kit (Covaris, Inc., Woburn, MA), as per manufacturer’s instructions with modifications. Post-extraction and purification, RNA was eluted in nuclease-free water, and yield was determined by the Quant-iT RNA HS Assay Kit (Thermo Fisher Scientific, Waltham, MA), as per manufacturer’s recommendations. A predefined optimal yield of 10ng RNA was used as specimen acceptance criteria to ensure adequate library generation, as per extensive analytical validation [2].

**Expression profiling.** The NGS assay utilizes the Oncomine Immune Response Research Assay (OIRRA) for gene expression profiling (Thermo Fisher Scientific). The panel uses multiplexed gene-specific primer pairs and NGS to amplify nucleic acids extracted from formalin-fixed paraffin-embedded (FFPE) slides. The complete OIRRA (covering 394 genes), as well as a subset adapted to quantify the expression of 54 validated target genes using 10 constitutively expressed housekeeping genes as normalizers, were utilized for analysis, as previously described [2]. OIRRA libraries were prepared using the Ion AmpliSeq targeted sequencing technology (Thermo Fisher Scientific) followed by enrichment and template preparation using the Ion Chef system (Thermo Fisher Scientific) and sequencing performed on the Ion S5XL 540 chip. Sequencing data were first processed using the Torrent Suite software (v5.2.0), during which previously-defined quality control (QC) specifications for mapped reads, on-target reads, and positive and negative expressed genes were used as sample acceptance criteria [2].

RNA-seq absolute reads were generated using Torrent Suite’s plugin immuneResponseRNA (v5.2.0.0). Absolute reads were further normalized to yield normalized reads per million (nRPM) using previously described methods [2]. For all 394 genes, nRPM values were subsequently ranked (gene expression Rank) from 0 to 100 based on expression of these genes in a reference population of 167 samples representing a wide range of gene expression in various tumor types. CD8 expression rank was interpreted as low, moderate and high based on cutoffs of the 25^th^ and 75^th^ percentiles.

**Statistical analyses.**

**Survival analyses:** For ICI-treated patients, survival was measured as time from date of first ICI treatment to death or last follow-up. In case of historical controls, survival was measured as time from date of date of biopsy proven Stage IV disease to death or last follow-up. Patients with less than 3 months follow-up time and vital status as alive were censored for this analysis. Five-year Kaplan-Meier survival curves were estimated using survfit function of Survival version 2.40.1 [3] library in R v.3.3.1. To test for statistical difference between the survival curves, post-hoc log-rank test p-values (Benjamini-Hochberg corrected) were calculated using pairwise_survdiff function of survminer library version 0.4.0. For all 110 cases with response, distribution of each biological function was split into 3 tertiles of low (less than 33), medium (between 33 and 66) and high (greater than 66). Next, we performed a pair wise proportion test (chi-square test) to test for difference in disease control rates for these three tertiles (i.e. low vs medium, medium vs high and low vs high) for each biological function. Proportion test was performed with continuity correction and pairwise p values for each biological function were adjusted for multiple hypothesis testing using “holmes” correction. We further divided the dataset into a training set (n = 76) consisting of samples from all data access groups except the largest contributor. A separate test set (n = 34) was constituted from samples from a single largest contributing institute. Any biological function that did not have cases representing one or more tertiles was removed from further analysis due to lack of dynamic range of that biological function in the population assessed in this study. The most significant gene functions were utilized for further analysis. Survival analysis was performed using a log-rank test on 5-year Kaplan-Meier survival curves for PD-L1 levels assessed by IHC and combined expression of 10 proliferation-related genes assessed by RNA-Seq. Comparison of DC rate was performed using Chi-squared test with Yate’s continuity correction. Multivariate analysis was performed by fitting a binomial logistic regression model to disease control labels and co-variates such as proliferation status, PD-L1 status, histology, race, sex, and age category (**Table S10**). Analysis of variance (ANOVA) was performed on the fitted model to study the table of deviance to determine the co-variate that explains the most variance in the disease control rates (**Table S11**).

**Supplementary Results**

**PD-L1 levels and proliferative status.** Survival analysis for PD-L1 as a positive result when TPS > 1% tumors showed very similar results to TPS >50% whereby moderately proliferative tumors with a median survival of 14.6 months was almost twice that of PD-L1 TPS <1% highly/poorly proliferative at 7.6 months (*p* = 0.2) (**Fig. S2**). There was a slight difference for median survival in moderately proliferative PD-L1 TPS <1% tumors at 12.6 months to that of highly/poorly proliferative PD-L1 TPS > 1% at 9.8 months (*p* = 0.62), but in both instances less than that of moderately proliferative PD-L1 TPS ≥ 1% tumors. Likewise, ICI-treated patients with PD-L1 TPS ≥ 1% tumors had a DC rate of 39.6% (21/53), whereas the rate was 24.6% (14/57) amongst individuals with PD-L1­ TPS <1% tumors (*p* = 0.1363) (**Fig. 5c**). Amongst moderately proliferative PD-L1 TPS ≥ 1% cases, the DC rate was 55.6% (15/27), which significantly higher than that of combined highly and poorly proliferative PD-L1 TPS ≥ 1% tumors (23.1%; 6/26; *p* = 0.0327), (**Fig. 5e)**. For PD-L1 TPS ≥ 1% poorly proliferative there were only seven cases for comparison (1 DC, 6 NDC). For PD-L1 TPS <1% tumors the difference in DC rate as compared to PD-L1 TPS ≥ 1% tumors in relationship to cell proliferation was significant. For moderately proliferative PD-L1 TPS <1% tumors the DC rate of 41.2% (7/17), was more than three times higher than that of poorly (17.6%; 3/17; *p* = 0.2588) or highly (17.4%; 4/23; *p* = 0.1911) proliferative PD-L1 TPS <1% tumors, or a combination of the latter two (7/40, 17.5%; *p* = 0.1179), (**Fig. 5g)**. The somewhat equal DC rates in moderately proliferative PD-L1 TPS ≥ 1% tumors (55.6%) to that in moderately proliferative PD-L1 TPS <1% tumors (41.2%) would support independence of these two biomarkers.

**References**

1. Eisenhauer EA, Therasse P, Bogaerts J, Schwartz LH, Sargent D, Ford R, et al. New response evaluation criteria in solid tumours: Revised RECIST guideline (version 1.1). Eur J Cancer. 2009;45:228–47. doi:10.1016/j.ejca.2008.10.026.

2. Conroy JM, Pabla S, Glenn ST, Burgher B, Nesline M, Papanicolau-Sengos A, et al. Analytical Validation of a Next-Generation Sequencing Assay to Monitor Immune Responses in Solid Tumors. J Mol Diagnostics. 2018;20:95–109. doi:10.1016/j.jmoldx.2017.10.001.

3. Therneau TM, Lumley T. Survival Analysis Guide. Cran. 2017;:143. doi:10.1016/j.jhydrol.2011.07.022.
